# Dissecting Interferon-Induced Transcriptional Programs in Human Peripheral Blood Cells

**DOI:** 10.1371/journal.pone.0009753

**Published:** 2010-03-22

**Authors:** Simon J. Waddell, Stephen J. Popper, Kathleen H. Rubins, Michael J. Griffiths, Patrick O. Brown, Michael Levin, David A. Relman

**Affiliations:** 1 Department of Medicine, Stanford University, Stanford, California, United States of America; 2 Department of Microbiology and Immunology, Stanford University, Stanford, California, United States of America; 3 Department of Biochemistry, Stanford University, Stanford, California, United States of America; 4 Howard Hughes Medical Institute, Stanford University, Stanford, California, United States of America; 5 Department of Paediatrics, Imperial College London, London, United Kingdom; 6 Veterans Affairs Palo Alto Health Care System, Palo Alto, California, United States of America; New York University, United States of America

## Abstract

Interferons are key modulators of the immune system, and are central to the control of many diseases. The response of immune cells to stimuli in complex populations is the product of direct and indirect effects, and of homotypic and heterotypic cell interactions. Dissecting the global transcriptional profiles of immune cell populations may provide insights into this regulatory interplay. The host transcriptional response may also be useful in discriminating between disease states, and in understanding pathophysiology. The transcriptional programs of cell populations in health therefore provide a paradigm for deconvoluting disease-associated gene expression profiles.

We used human cDNA microarrays to (1) compare the gene expression programs in human peripheral blood mononuclear cells (PBMCs) elicited by 6 major mediators of the immune response: interferons α, β, ω and γ, IL12 and TNFα; and (2) characterize the transcriptional responses of purified immune cell populations (CD4^+^ and CD8^+^ T cells, B cells, NK cells and monocytes) to IFNγ stimulation. We defined a highly stereotyped response to type I interferons, while responses to IFNγ and IL12 were largely restricted to a subset of type I interferon-inducible genes. TNFα stimulation resulted in a distinct pattern of gene expression. Cell type-specific transcriptional programs were identified, highlighting the pronounced response of monocytes to IFNγ, and emergent properties associated with IFN-mediated activation of mixed cell populations. This information provides a detailed view of cellular activation by immune mediators, and contributes an interpretive framework for the definition of host immune responses in a variety of disease settings.

## Introduction

Interferons are a class of cytokines first identified in 1957 as having a protective effect against viral infection [1]. Interferons can be divided into three groups; type I (IFNα/β/ε/κ/ω) that engage the IFNAR1/2 receptor, type II (IFNγ, the sole member) that signal through the IFNGR1/2 receptor [2], and type III (IFNλ) that utilize IFN-λR1 and IL-10R2 receptors [3], [4].

The type I interferons, IFNα (of which there are 13 subtypes), IFNβ and IFNω are secreted by most cell types in response to viral infection [5]. Mice lacking intact interferon receptors are highly susceptible to viral infection [6]. Type I IFN stimulation induces a number of different systems involved in the activation of the immune response, cell growth and the control of apoptosis, in addition to the PKR (dsRNA-dependent protein kinase), 2-5A synthetase and Mx antiviral systems [7], [8]. Type I interferon subtypes have also been reported to have distinct activities [9], [10]; these IFN subtype-specific effects are influenced by factors such as receptor binding efficiencies [11], constitutive levels of IFN expression [12], and the specific viral-target cell combination [13]. By contrast type II interferon (IFNγ), secreted by activated NK cells and T lymphocytes, has been implicated primarily in the activation of macrophages and has been demonstrated to be important for the protection of the host against intracellular pathogens such as *Leishmania*, *Toxoplasma* and *Mycobacterium* species [14]. Mutations in the IFNγ receptor have been associated with increased susceptibility to mycobacterial infection [15]. Interferons are involved in a wide range of clinically important phenomena, ranging from activation of immune responses to infection [14] to cancer suppression [16] to depression [17]. Recombinant interferon therapy has been approved for a spectrum of conditions such as hepatitis B and C infections, Kaposi's sarcoma, multiple sclerosis and chronic granulomatous disease [5].

DNA microarray analysis of gene expression has enabled the description and discrimination of disease states [18]–[21]; and presents an opportunity for both diagnostic and prognostic marker discovery [22]–[24]. IFN signatures have been identified as prominent aspects of many transcriptional profiles [25]–[28]. However, to interpret these gene expression patterns further, a basic understanding of the response of complex cell populations to various stimuli is required. Microarray analyses have been used previously to investigate the global effects of interferon stimulation in human non-immune cells after 6 h incubation in a fibrosarcoma cell line [29], in murine fibroblast cells [30], in primary endothelial cells after 18 h treatment [31], and in epithelial cells using ChIP-chip technology to investigate STAT1/STAT2 binding events [32]. An increased understanding of the temporal and cell-specific nature of gene expression in response to cytokine stimulation may reveal insights into the activation and interactions of different cell types during infection.

In this study we used human cDNA microarrays (1) to compare the responses of a mixed population of immune cells (human peripheral blood mononuclear cells) to stimulation with 6 major mediators of immune activation – the human type I interferons (IFNα, β and ω), type II interferon (IFNγ), and two factors involved in cell-mediated immunity (IL12 and TNFα); and (2) to contrast the transcriptional reorganization of purified immune cell populations (CD4^+^ and CD8^+^ T cells, B cells, NK cells and monocytes) to treatment with IFNγ. This gene expression analysis of both mixed and purified immune cell populations sampled over time allowed both cytokine-specific and cell-specific transcriptional patterns to be identified. This information may assist in the recognition of host immune responses in disease settings, help to reveal novel immuno-modulatory actions of pathogens during infection, and offers a global view of cellular activation after exposure to a variety of cytokines.

## Results

### PBMC cytokine activation profiles

**Figure 1:**
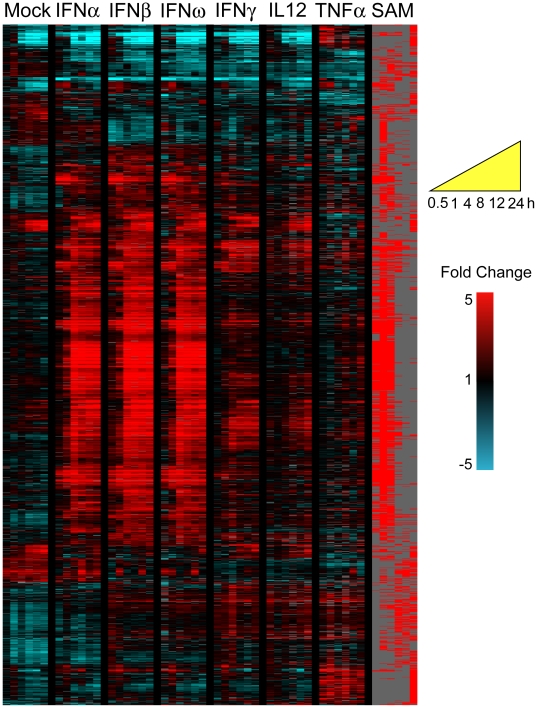
PBMC transcriptional programs elicited by cytokine exposure. The PBMC response to stimulation with 0.6 pM IFNα, β, ω, γ, IL12 and TNFα from 30 minutes to 24 h after treatment. 1857 genes were identified to be significantly differentially expressed in response to one or more stimuli compared to mock-treated PBMC (0.1% BSA/PBS). The expression profiles are ordered by hierarchical clustering; the genes are displayed as rows, time points/conditions as columns, in temporal order (see yellow key). Red coloring signifies the up-regulation of expression; blue coloring denotes down-regulation. Samples were taken at time intervals of 0.5, 1, 4, 8, 12 and 24 h. The column marked SAM indicates (in red) which genes were identified to be significantly differentially expressed in response to each cytokine.

To investigate the temporal program of gene expression in a physiologically relevant mixed cell population specific to activators of innate immunity, we treated PBMCs with recombinant IFNα2b, IFNβ1a, IFNω, IFNγ, IL12 and TNFα and sampled at intervals of 0.5, 1, 4, 8, 12, and 24 h post stimulation. Differentially expressed genes were identified using the Significance Analysis of Microarrays (SAM) algorithm to compare the temporal response of each treatment with a mock time course (treatment with 0.1% BSA/PBS), as well as to all other treatments. The temporal expression pattern of 1857 genes found to be significantly differentially expressed (FDR<1%, minimum of 2 fold change) are shown in Figure 1. Tables 1 and 2 detail the numbers of genes differentially expressed by each treatment relative to the mock time series. The magnitude of differences in transcript abundance, and the correlations between responses to each pair of stimuli are detailed further in Tables S1 and S2. No significant changes were detected by flow cytometry in the proportion of major cellular subsets in PBMCs over the 24 h time period, confirming that differences in gene transcript abundance were due to changes in transcript abundance and not to shifting demographies of PBMC sub-populations.

**Table 1:** PBMC transcriptional responses to 0.6 pM IFNα, β, ω, and γ, IL12 and TNFα.

	IFNα	IFNβ	IFNω	IFNγ	IL12	TNFα
Induced	226	370	288	111	57	130
Repressed	31	114	89	77	53	125

Numbers of genes are indicated. Differentially expressed genes were identified by SAM as significantly more highly expressed or under-expressed relative to mock treated time series.

**Table 2:** PBMC common type I IFN transcriptional program, and common IFNγ and IL12 program.

	Stereotyped type I IFN response	IFNγ-IL12 response
Induced	201	35
Repressed	17	35

Numbers of genes are indicated. Differentially expressed genes were identified by SAM as significantly more highly expressed or under-expressed relative to mock treated time series.

The human immune cells were treated with cytokines at a standardized concentration of 0.6 pM, in order to facilitate comparisons among the cytokine activation profiles; the use of units (U) was undesirable in this setting as these values are calculated with biological assays, and are based on different parameters for each cytokine. However, the influence of cytokine concentration on the pattern of transcription was addressed in an additional experiment, in which PBMCs were treated with 0.006, 0.6 and 60 pM of recombinant IFNγ (corresponding to 1, 100 and 10,000 U IFNγ, respectively) and sampled at 0.5, 1, 2, 4, 8 and 12 h post stimulation (Figure S1). The qualitative transcriptional responses were similar for the three IFNγ concentrations, but the amplitude of the responses increased with IFNγ dose. The subset of genes differentially induced by type I, as opposed to type II IFN, was not induced even at the highest tested concentrations of IFNγ; suggesting that the differential responses of PBMCs to type I and type II interferons cannot be explained by a difference in effective dose.

### A common stereotyped response to type I IFNs

A common activation profile of 201 genes (Table S3) was induced in a stereotyped temporal pattern by the type I interferons (significantly more highly expressed by IFNα, IFNβ and IFNω treatment compared to mock); the consistency of this response is reflected by the high correlation scores between type I IFN treatments (Table S1). The similarity between gene expression responses to IFNω and IFNα/β confirms that these interferons trigger similar physiological events, and are likely to signal through the same type I interferon receptor [7]. Genes encoding proteins implicated in the major antiviral pathways initiated by interferon stimulation were consistently induced, including the dsRNA-dependent protein kinase pathway (PRKRA, EIF2AK2 (PRKR), PALM2-AKA1, EIF2B1), the 2-5A synthetase system (OAS1/2/3), and the Mx pathway (MX1/2). Genes involved in the control of apoptosis were also highly expressed after type I IFN treatment (CASP1/5/10, FAS, FASLG, FAF1, GADD45B, BAG1). Type I interferon stimulation also led to increases in transcripts encoding a range of immune cell surface receptors - CD4, CD38, CD69, CD2AP, IL15RA, IL8RB, LEPR, MSR1, TLR3, TLR7, TNFRSF11A, TRD. The induction of TLR3 (recognizing dsRNA) and TLR7 (recognizing ssRNA) suggests enhanced vigilance against viral infection activated by type I IFNs (MYD88 was also up-regulated). The stimulation of human PBMCs with type I IFNs also induced a number of genes encoding soluble factors such as the chemokines, CCL7, CCL8, CCL13, CXCL9 (MIG), CXCL10 (IP10), CXCL11 and the interleukins, IL6 and IL15. Genes encoding components of the IFN signaling pathways, including the transcriptional mediators STAT1 and STAT2, the Janus activated kinase JAK2, and the negative regulator of JAK-STAT signaling SOCS1 (SSI-1) [33] were consistently highly expressed; together with a number of genes encoding transcription factors ATF3/5, IRF2/7, IFI16, SP100/110, SPIB, TFDP2. Numerous transcripts encoding proteins whose roles remain to be fully characterized were also significantly induced G1P2, G1P3 (6–16), IFI27, IFI35, IFI44, IFIT1/5, IFITM1 (9–27), IFITM2/3, IFRD1, IFRG28, ISG20, as previously described [14].

### Type I vs. type II IFN programs

**Figure 2:**
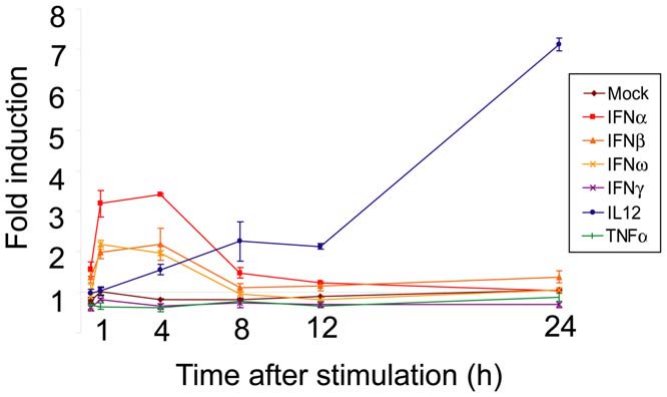
Induction of IFNγ in PBMCs after IL12 exposure. The mean expression profile of IFNγ transcripts after exposure of PBMCs to 0.6 pM IFNα, β, ω, and γ, IL12 and TNFα; as determined by microarray analysis at time intervals of 0.5, 1, 4, 8, 12 and 24 h post stimulation. Standard deviations are indicated with error bars.

IFNγ signals through a different specific receptor and has biological effects distinct from those of the type I interferons *in vitro* and *in vivo* [34]. The transcriptional response of PBMCs to IFNγ stimulation in the initial 24 h appears largely to be restricted to a subset of type I IFN-inducible genes (Figure 1). This statement must be qualified with the observation that transcripts of IFNγ itself were induced within the first 4 h of type I IFN treatment (Figure 2), which may result in the initiation of an IFNγ transcriptional program in type I IFN-treated cells. IFNα has also been demonstrated to promote the proliferation of IFNγ-secreting T cells [35].

We identified 114 genes that were significantly more highly induced by all type I IFNs than by IFNγ treatment, and 10 genes that were more highly induced by IFNγ stimulation (in a direct comparison between type I and II IFN responses, Table S4). As expected, genes associated with the anti-viral PKR, 2-5A synthetase and Mx pathways were more highly induced by type I IFNs, correlating with the greater antiviral potency of type I compared to type II IFNs [5]. The interferon regulatory factors IRF2 and IRF7, and the transcription factors ATF3, ELF1, IFI16, KLF6, OLIG2, SP100, SP110, and SPIB were also consistently more highly expressed by type I than by type II IFNs. The 10 genes more highly induced by IFNγ than by type I IFN treatment included FCGR1A which encodes a high affinity Fc receptor, the IL6 receptor (IL6R), the chemokine CXCL9, the macrophage lectin (CLECSF14), ubiquitin D (UBD), the transcriptional enhancer C/EBPα and the MHC class II regulator MHC2TA (CIITA). MHC2TA has previously been noted to be induced to a greater degree by IFNγ treatment than by type I IFN treatment [14].

### Parallels between transcriptional responses to IFNγ and IL12

Stimulation with IFNγ resulted in the induction of 111 genes, including the chemokines CCL8, CXCL9, CXCL10 and CXCL11 and the interleukins IL1A, IL7 and IL15, together with the cell surface receptors CCR5, CD38, CSF2RB, FCGR1A, ICAM, IL15RA, MSR1, NKTR, P2RY13, SLAMF1. IFNγ stimulation also induced several factors involved in the complement system (BF, C1QB, C4A and SERPING1). Additionally, genes implicated in the metabolism of tryptophan (INDO, WARS) and MHC class I processing (PSMB9, TAP1/2) were highly expressed after treatment with both type I and type II IFNs. The transcriptional response of PBMCs to IL12 stimulation was similar to the IFNγ activation program, as evidenced by the high correlation score and small number of differentially expressed genes that distinguished these two treatments (Table S1). Interestingly the transcriptional response to IL12 appeared to be delayed by approximately 4 h when compared to the response to IFNγ (Figure 1). IFNγ-induced genes were significantly enriched in the subset of genes up-regulated by IL12 (hypergeometric p value  = 5.6×10^−4^). Transcripts of IFNγ were significantly induced by IL12 treatment (Figure 2), as were IFNγ levels in the culture medium (data not shown). The delayed IFNγ-like response following IL12 treatment may therefore be due to the transcriptional induction and subsequent release of IFNγ from IL12-activated T cells in the mixed cellular population of PBMCs. The similarity in PBMC gene expression patterns resulting from IFNγ or IL12 stimulation may also reflect the induction of analogous pathways involved in macrophage activation, and explain why polymorphisms in IFNγ and IL12-related genes often result in similar pathological conditions [15], [36].

### Transcriptional signature of TNFα

The response to TNFα was strikingly different from all of the IFN response programs (Figure 1). The 130 genes induced by TNFα treatment of PBMCs encode cytokines and chemokines implicated in the orchestration of the inflammatory response (CCL15/20/23, CCL3L1, CXCL1/2/3, IL16, IL18, IL1A, IL1B, IL6, IL8, SLAMF1, TNF), and a number of genes involved in NFKB activation and its regulation (IKBKG, NFKB1, NFKB2, NFKBIA, NFKBIZ, RELB, TNFAIP2/3, TNFRSF11A, TRAF1). The gene ontology terms associated with this group were significantly enriched for functional categories such as cytokine activity, response to external stimulus, and NFKB signaling. In addition, functional groups involved in chemotaxis, and the regulation of cell proliferation or apoptosis were also associated with the genes induced by TNFα treatment. The activation of the NFKB pathway is a well-established consequence of TNFα ligation [37], [38], and was not associated with the gene ontology terms linked to IFN stimulation.

### Cell-type associated gene expression

**Figure 3:**
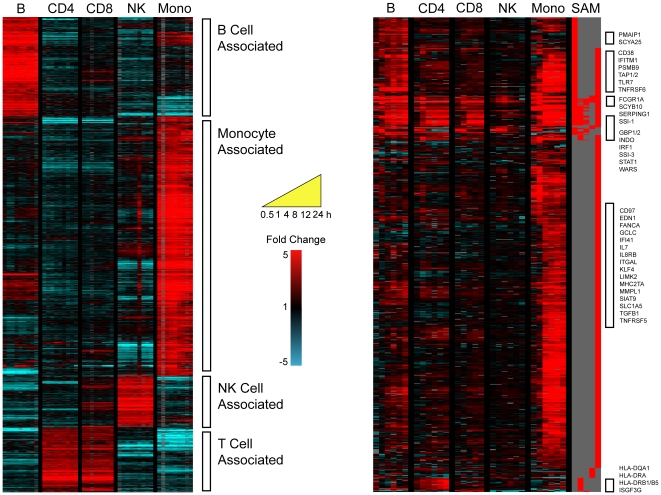
Cell-type associated gene expression. **A (left)**. Cell type-associated genes identified from the transcriptional profiles of unstimulated isolated cell types (time zero and 0.1% BSA/PBS-treated time series). Genes were defined as cell type-associated if they were identified as significantly more highly expressed in a single cell type compared to all other cell types. The expression profiles are ordered by hierarchical clustering; the genes are displayed as rows, cell type/time points as columns. Red coloring signifies high expression; blue coloring denotes low expression. Cell type-associated gene clusters are marked. **B (right).** The cell type-specific nature of IFNγ transcriptional responses. Purified subsets of B cells, CD4^+^ T cells, CD8^+^ T cells, NK cells and monocytes were stimulated with 0.6 pM IFNγ and sampled at 0.5, 1, 4, 8, 12 and 24 h. 807 significantly induced genes were identified, and ordered by cell type and hierarchical clustering. The genes are displayed as rows, time points/cell subsets as columns. Red coloring signifies up-regulation, and blue coloring signifies down-regulation of expression after IFNγ exposure relative to the mock-treated discrete cell population. The column marked SAM indicates (in red) which genes were significantly differentially expressed by IFNγ in each cell type. Genes of interest are marked as annotated in Source [73].

To determine which cell types present in PBMCs contributed to the IFNγ signature described above, and to investigate whether discrete subpopulations of immune cells respond in the same way to stimuli as do mixed populations, CD4+ T cells, CD8+ T cells, B cells, NK cells and monocytes were each isolated from human PBMCs and then stimulated with IFNγ. The reliability of this cell separation and microarray analysis strategy was confirmed by comparing the gene expression profiles of the unstimulated cell subsets (time zero and 0.1% BSA/PBS treated time points). Genes were defined as cell type-associated if they were identified (by multiple SAM two class pair wise comparisons, FDR<1%, minimum of 2 fold change) as significantly more highly expressed in a single cell type when compared to all other cell subsets (Figure 3a). Through this procedure, 179 genes were determined to be B cell associated, 210 genes were T cell associated (with 17 CD4+ and 25 CD8+ associated genes); 161 genes were NK cell associated and 1042 were monocyte associated (Table S5). The expression of known cell type-specific genes (such as CD4, 8, 14, 19 and HLA molecules) was restricted to the appropriate purified cell subset. In addition, the match between the B cell and T cell specific genes found here and in a previously published study was highly significant (hypergeometric probability 5.68×10–149, and 5.87×10–74 respectively) [39].

### Cell-type specific IFNγ responses

To investigate the immune cell specificity of activation programs induced by a major component of cell-mediated immunity, the responses of these isolated cell populations to 0.6 pM (100 U) IFNγ was measured at 0.5, 1, 4, 8, 12 and 24 h. Significantly induced genes were identified by comparing each time series to a parallel unstimulated mock time course of the same purified cell type (FDR<1%, minimum of 2 fold change). 80 genes were significantly up-regulated in B cells by IFNγ stimulation, 36 in CD4+ T cells, 21 in CD8+ T cells, 17 in NK cells and 294 in the purified monocyte population (Figure 3b and Table S6). Six genes were significantly induced in all cell types (B, T, NK lymphocytes and monocytes): those encoding the chemokine CXCL10 (SCYB10); the complement regulatory peptidase inhibitor SERPING1; the suppressor of cytokine signaling SOCS1 (SSI-1); ribosomal protein RPS9; the niacin receptor 2 HM74 (NIACR2); and the high affinity Fc receptor FCGR1A (CD64). The transcriptional activators STAT1 and IRF1 were significantly induced in CD4+ and CD8+ T cells, B cells and monocytes; while ISGF3G (interferon-stimulated transcription factor 3 gamma, IRF9) was found to be significantly up-regulated in only CD4+ T cells. Genes coding for the MHC class II proteins HLA-DRA, B1, B5 and HLA-DQA1 were highly expressed in CD4+ cells. Expression of class II MHC has been previously demonstrated in non-professional antigen-presenting cells after IFNγ stimulation, and in activated T cells [40]. The B cell response to IFNγ included the induction of genes encoding CD38 and CD69, both of which have been implicated in B cell activation and maturation, and a number of genes involved in MHC class I presentation such as the proteasome subunits (PSMB8 and B9, PSME1 and 2) and TAP1 and 2 transporters. Interestingly, neither STAT1 nor IRF1 were significantly induced as part of the limited transcriptional response of purified NK cells to IFNγ.

As expected, monocytes had the greatest response to IFNγ stimulation; activation of macrophages is a well-defined role of IFNγ. Of the 294 genes that were induced by IFNγ (61 of which were identified to be monocyte-associated), over 40 coded for cell surface molecules such as the chemokine/cytokine receptors (CCRL2, CRLF1, CSF2RB, HM74, IL15RA, IL2RA, IL6R, IL8RB), the cell activation markers (CD36, CD38, CD69, CD97), and a number of proteins involved in cellular adhesion (CD226, EVA1, ICAM1, ITGAL, ITGA4, ITGB7, LGALS3BP, MUC1, SIAT1). The up-regulation of numerous genes involved in MHC class I and II expression (TAP1 and 2, TAPBP, MHC2TA, RFX5, HLA-DMA/B, HLA-DNA, HLA-DPB1), proteasome formation (PSMA2, 4, and 5, PSMB8, 9, and 10, PSME1 and 2) and protein turnover (specifically ubiquitination - UBD, UBE2E2, UBE2L6, UBE3A, USP25, LOC51619) underscores the role of IFNγ in promoting antigen processing and presentation in monocytes. The major mediators of IFNγ-induced signaling, STAT1, JAK2, and IRF1, were induced together with several factors that may regulate STAT and JAK function (CISH, NMI, PTPRC, PTPRO, SOCS1) [34]. IFNγ stimulation also resulted in the increased expression of an additional 32 proteins predicted to regulate transcription, including C/EBPα, EGR2, HLX1, IFI16, IRF8, KLF2 and 4, STAT2, SP110 and the MHC II regulatory elements MHC2TA and RFX5. In a complementary approach, putative C/EBP, EGR, HOX, IRF, ISRE, MEF and STAT transcription factor binding sites were amongst 104 motifs identified after searching 4000 nt either side of the start sites of the 294 genes induced by IFNγ treatment in the purified monocyte population. The integration of temporal patterns of gene expression with transcription factor motif mining tools promises to reveal novel regulatory networks affecting immune cell function [41].

### Temporal response to IFNγ

**Figure 4:**
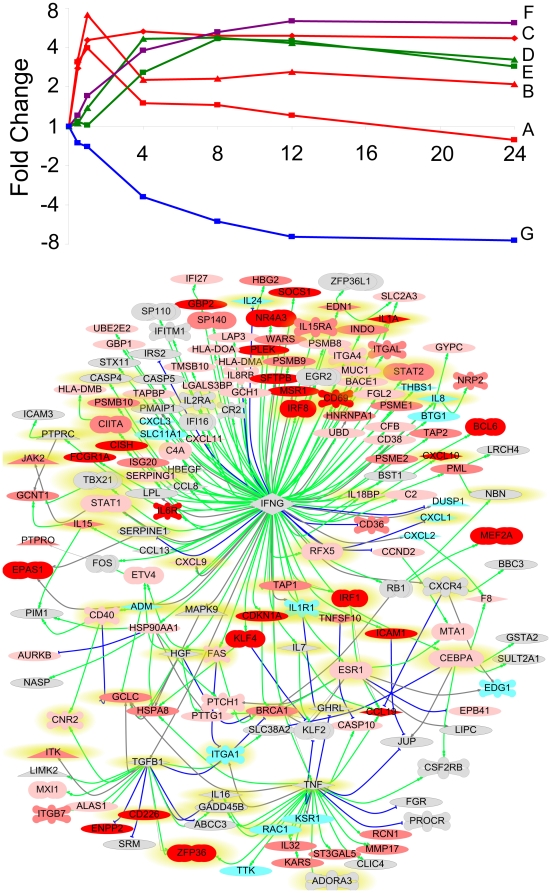
Monocyte responses to IFNγ. **A (top).** The temporal transcriptional responses of monocytes to IFNγ stimulation. Of 363 genes that were significantly differentially expressed following treatment with 0.6 pM IFNγ, 264 genes were assigned to 7 significantly represented expression profiles, A–G. The temporal response (measured in mean fold change) of the genes assigned to each cluster is plotted at 0.5, 1, 4, 8, 12 and 24 h post treatment. Clusters A (square), B (triangle) and C (diamond) colored red; D (triangle) and E (square) in green; F (square) in purple; and G (square) in blue. **B (bottom).** A network of predicted crosstalk downstream of IFNγ exposure. Previously characterized interactions between IFNγ and the 363 genes identified as differentially expressed after IFNγ treatment in monocytes were mapped. The expression of 109 genes were predicted to be directly affected by IFNγ. Secondary regulatory events were characterized by the expression of 65 additional genes (identified to be differentially expressed by IFNγ treatment) whose expression was modified by those 109 genes but not by IFNγ directly. This network illustrates the complexity of possible interplay downstream of IFNγ stimulation. In addition, 61 proteins reported to have an effect on IFNγ expression itself are highlighted in yellow, and may represent positive and negative feedback loops mediating cell activation. Gene identifiers are colored by temporal expression pattern after IFNγ treatment as detailed in Figure 4A (dark red (profiles A–C), lighter red (D and E), pink (F), blue (G), grey (unassigned)). Gene identifiers are also shaped by putative function, transcription factors (ellipse), kinase/phosphatases (triangle), ligands (rhombus), receptors (cross). The nature of the interactions are indicated with connecting lines either reflecting a positive (green), negative (blue), or undefined (grey) impact on downstream gene or protein expression.

The temporal response of the monocyte population to IFNγ treatment was analyzed by partitioning the differentially expressed genes into 7 significantly represented expression profiles using Short Time-series Expression Miner (STEM) [42] (Figure 4a, detailed in Table S7). Correlating the temporal pattern of monocyte gene expression with the enriched functional categories of genes may help to elucidate the timing of events following monocyte activation. For example, the functional category for the proteasome complex was highlighted in profiles of induced genes with peak transcript abundance at 4 h and 8 h after IFNγ treatment (Figure 4a, profiles D and E); perhaps reflecting a time-delayed transcriptional response after signaling cascades were triggered by IFNγ receptor cross-linking. The GO ontology terms for chemotaxis (and taxis) were significantly enriched in the subset of genes repressed after IFNγ stimulation (Figure 4a, profile G); which may be an indicator of monocyte activation and differentiation.

Most interestingly, the functional category associated with negative regulators of cellular or biological processes was significantly enriched among genes induced within 1 h of IFNγ treatment (Figure 4a, profiles A–C). 15 genes shared this gene ontology term, including BCL6 (a zinc finger transcriptional repressor) [43], and two suppressors of cytokine signaling, SOCS1 a pseudosubstrate inhibitor of JAK-STAT signaling [44], and CISH (CIS/SOCS) an inhibitor of growth hormone signaling through STAT5b [45]. The up-regulation of CISH together with the significant enrichment of genes containing STAT5 binding motifs induced within 1 h of IFNγ stimulation (Figure 4a, profiles A–C) predicts that monocyte activation is likely to be partially mediated through STAT5 (even though STAT5 itself was not identified in this analysis as significantly differentially expressed). RHOH, a repressor of Rac GTPase-mediated signaling (specifically RAC1) [46], was also induced immediately after IFNγ treatment; conversely, RAC1 was repressed with time (Figure 4a, profile G). RHOH has recently been demonstrated to down-regulate leukotriene production in neutrophils [47], and may therefore fulfill a similar negative feedback function during monocyte activation. Tristetraprolin (TTP, ZFP36), induced within 1 h by IFNγ (Figure 4a, profile A), has been implicated in the rapid degradation of IFNγ and IL2 mRNA [48] and may therefore play an important role in constraining the monocytic pro-inflammatory response. Other interactions that may temper the activation state of monocytes after IFNγ stimulation include the repression of pro-inflammatory ligand receptors, IFNγ receptor 1 (IFNGR1, although this was not identified by the significance testing algorithm), the IL1 receptor 1 (IL1R1) [49], and chemokine receptor (CXCR4). The induction of CISH and RHOH and the repression of IFNGR1 in monocytes after IFNγ exposure were confirmed by quantitative RT-PCR, as was the modest induction of STAT5A (Figure S2).

By modeling the monocyte response over time, possible signaling cascades could be recognized; for example, the generation of MHC class II molecules is dependent on the expression of the class II transactivator CIITA (MHC2TA) that is in turn induced by IRF-1 [50]. This pathway, resulting in MHC class II molecule expression after IFNγ stimulation, can be followed through the time series with the induction of IRF-1 within minutes of IFNγ treatment (Figure 4a, profile C), followed by the up-regulation of CIITA peaking at 4 h (Figure 4a, profile D), and then the steady induction of MHC class II genes (HLA-DMA, DMB, DOA, DPB1, Figure 4a, profile F). In an example of negative regulation, the subset of genes repressed after IFNγ treatment (Figure 4a, profile G) was significantly enriched for AP-1 binding sites. This (together with the down-regulation of FOS itself) may reflect the action of BCL6, a transcriptional repressor that has been demonstrated to block AP-1 activity [43], which is induced with a peak at 1 h after IFNγ stimulation (Figure 4a, profile B). The complexity of potential crosstalk following monocyte stimulation by IFNγ is summarized in Figure 4b; where the previously characterized interactions (identified in the ResNet 6 Mammalian database) downstream of IFNγ are mapped onto the genes identified in this study to be differentially expressed by IFNγ treatment. Proteins whose expression is altered by IFNγ, that may also have a regulatory effect on IFNγ expression itself, are highlighted in yellow.

### Transcriptional plasticity of immune cells to stimulation

**Figure 5:**
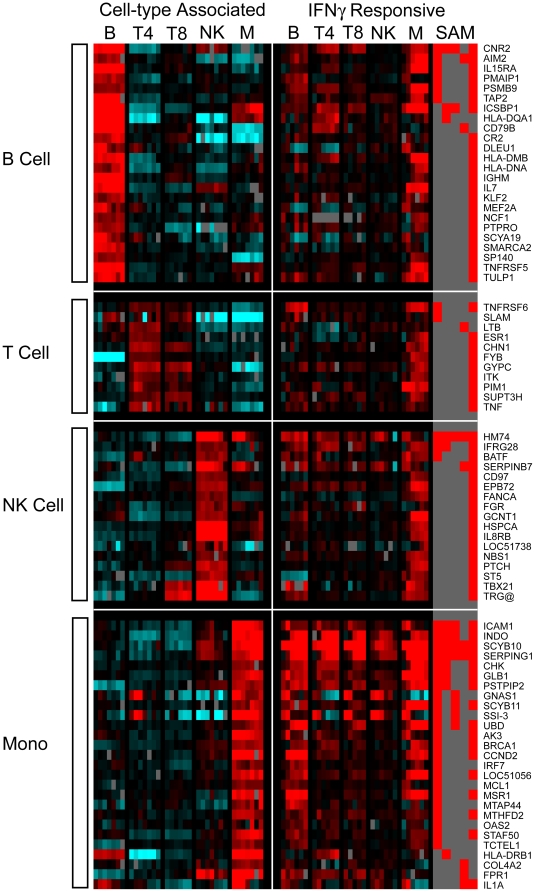
The transcriptional plasticity of immune cellular responses to IFNγ stimulation. 79 genes associated with a single isolated immune cell subtype (without stimulation) were induced after IFNγ treatment in other cell types. Genes defined as cell type-associated (by comparing unstimulated time series, as detailed in Figure 3A) are marked on the left. The top panel detailing B cell associated genes, followed by T cell, NK cell, and monocyte associated genes illustrated in the bottom panel. The differential regulation of these genes after IFNγ treatment in each cell type (as in Figure 3B) is described on the right. The expression profiles are ordered by hierarchical clustering; the genes are displayed as rows, cell type/time points as columns. Red coloring in the cell-type associated panels (on left) signifies highly expressed in a single cell type compared to all other cell types; blue coloring denotes low expression. Red coloring in the IFNγ responsive panels (on right) signifies up-regulation, and blue coloring signifies down-regulation of expression after IFNγ exposure relative to the mock-treated discrete cell population. B (B cells); T4 (CD4^+^ T cells); T8 (CD8^+^ T cells); NK (NK cells); M (monocytes). The column marked SAM details (in red) which genes were significantly differentially expressed by IFNγ in each cell type. Genes are marked as annotated in Source [73].

This transcriptional analysis of immune cell subtype responses to IFNγ treatment allowed us to investigate the effect of immune stimulation on the expression of cell-associated signatures. The changing RNA abundance profiles of these cell type-associated genes were revealed by comparing the cell type-associated gene expression signatures (Figure 3a, Table S5) with the cell subset responses to IFNγ stimulation (Figure 3b, Table S6). 79 genes, identified as cell type-associated in unstimulated discrete populations, were recognized to be induced by IFNγ treatment in a different cell subtype population (Figure 5). After IFNγ stimulation, these genes were no longer expressed in a cell type-specific manner; moreover, these genes were not necessarily IFNγ-inducible in their associated cell type. The term transcriptional plasticity has been adopted in this setting to describe the regulation of cell type-associated genes in other cell subsets after stimulation. For example, MHC Class II (HLA-DMB/DNA/DQA1, TAP2) and immunoglobulin genes (CD79B, IGHM) associated with B cell gene expression were induced after stimulation in other cell types; of these genes, only TAP2 was induced by IFNγ treatment in the discrete B cell population. The transcriptional flexibility of immune cells following stimulation, with the differential expression of cell type-associated genes in distinct isolated cell populations, suggests a plasticity of the cellular immune response that deserves further attention.

### IFNγ-induced transcripts unique to mixed-cell populations

The overlapping transcriptional signatures of cells stimulated with IFNs is both cell subset- and IFN-specific; for example, STAT2, an important mediator of type I interferon signaling, was induced by IFNγ treatment only in the purified monocyte cell population, as were an additional 22 genes induced by type I IFNs, compared to IFNγ stimulation in PBMCs (Table S4). We did not detect changes in relative abundance of transcripts in the IFNγ-treated PBMCs for many of the genes whose transcripts were detected as induced by IFNγ in the purified monocyte population, which was possibly a reflection of the dilution of monocyte-specific transcripts in the total PBMC cellular RNA population. More interestingly, 40 genes (including CCR5, CD83/86, GOS2, INHBA, IRF4, OAS1/3, CCL2/3/4/7, SELPLG and TNFAIP6, Table S8) that were significantly induced by IFNγ in the heterogeneous population comprising PBMCs were not detected as induced to a significant degree in any of the purified cell subsets. 15 of these genes were identified to be part of the TNFα activation program (Figure 1) and 22 genes were monocyte-associated (Figure 3a), suggesting that monocytes may respond to additional interactions within the heterogeneous PBMC population. This rudimentary analysis highlights the emergent physiology of a tissue, in this case, peripheral blood, comprising a mixture of communicating cells.

## Discussion

We characterized the differential effects of 6 cytokines on gene expression in a mixed population of human immune cells; defining for the first time the conserved transcriptional response to IFNω, together with IFNα and β, and revealing major similarities in IFNγ and IL12 transcriptional programs. We and many others have examined gene expression profiles in peripheral blood *in vivo* and *ex vivo* in order to identify both gene expression signatures and specific regulatory pathways activated in health-associated homeostasis and in disease. Data derived from carefully controlled experiments establishing the transcriptional response to specific stimuli in relevant cell populations have become an important part of the framework used to interpret patterns of gene expression observed in these complex studies. The response to interferons has gained a prominent central role in the study of many immune processes. We have observed an interferon-response gene expression signature associated with shock in dengue infection [26], with early pre-clinical responses to Ebola [51], with the response of leukocytes to *Neisseria meningitidis* [52], and with a pattern of gene expression that discriminates Kawasaki Disease and acute adenovirus infection [53]. The detailed description of the shared and unique features of the transcriptional programs of the cytokines used in this study, and of the contribution of individual cell types to the gene expression patterns observed in a mixed cell population constitutes a valuable resource that has facilitated our analysis of these previously-published data sets, and will enable researchers to better delineate regulatory networks, elucidate pathways that are disrupted during disease, and deconvolute gene expression patterns in cell populations exposed to multiple complex stimuli.

Other groups have examined the response to interferons in non-immune cell populations. Many of the observations of Der *et al*. [29] who investigated the expression profiles of HT1080 cells (a human fibrosarcoma cell line) after 6 h stimulation with interferons IFNα, β, and γ by microarray analysis, were confirmed in this study. A core set of established IFN-regulated genes were identified by both analyses; however, there are subsets of genes that appear to be induced differentially in these two studies. This may be due to technical differences such as concentration of interferons, microarray platform and gene annotation, but may also reflect cell-specific (professional immune vs non-immune) responses to IFN stimulation [54]. It is also possible that differences reflect donor-to-donor variation in interferon-mediated responses. However, the overall similarity in findings, in addition to the results from studies investigating inter-individual variation in gene expression patterns, indicate that the stimulus rather than a donor-specific background is likely to be the primary feature driving transcriptional responses of peripheral blood immune cell populations [22], [55]–[57]. Similarly, comparison of our results with those from studies of IFNγ stimulation of primary endothelial cells and HT1080 cells [31], [58] revealed broad concordance, with a few exceptions. A recent study by Indraccolo *et al*. [59] identified 41 genes to be more highly induced by IFNα compared to IFNγ in human umbilical vein endothelial cells; as in our study, the majority of these genes were predicted to be involved in antiviral responses. Six genes were induced to a greater degree by IFNγ than by IFNα; of these, CXCL9 (MIG) and ubiquitin D (UBD or FAT10), both of which are involved in targeting proteins for proteasomal degradation [60], were also identified in the present study. Therefore, the chemokine, CXCL9, and UBD, which is a marker of immune activation in hepatocellular carcinoma and colon carcinoma [61], are preferentially induced by type II interferon in both professional immune and non-immune cells.

A parallel analysis of IFNγ responses of isolated subsets of the cells comprising the PBMC pool allowed us to begin dissecting the contribution of specific cells in this complex tissue. The transcriptional response to IFNγ in T cell, B cell and NK cellular populations was limited in comparison to the large number of genes induced in purified monocytes, where RHOH and CISH are predicted to have novel regulatory roles in controlling monocyte activation. These transcript abundance datasets may be utilized in combination to examine which particular cell types are responding to specific stimuli in dynamic scenarios. For example, the transcription factors C/EBPα, involved in myeloid cell differentiation [62] and associated with acute myeloid leukemias [63], and MHC2TA (CIITA), the major regulator controlling expression of MHC class II molecules [50], were both preferentially induced by type II IFN (in PBMCs compared to type I IFNs and TNFα). These genes were also up-regulated with time in the monocyte population alone (in response to IFNγ treatment), suggesting that the expression of these major mediators of cell activation in a complex model of infection may be due primarily to the response of monocytes to IFNγ. This study also highlighted the plasticity of the cellular transcriptional response to stimulation, with the induction of ostensibly cell type-specific genes in alternative cell types after treatment with IFNγ. Comparison of the expression patterns derived from mixed and purified immune cell populations after IFNγ treatment illuminated differences in the way in which cells responded to a stimulus in isolation versus a complex environment, with a number of genes only induced by IFNγ stimulation in complex cellular populations. The immune response to pathogens requires the coordinated interaction of multiple cells, and the presence of different cell populations; thus, the cross-talk between populations may be more representative of *in vivo* functioning than are the findings from isolated cell populations. Further investigation into these interactions may provide novel insights into cellular activation and complex responses measured from mixed populations.

The focus of this study has been on the defined actions of cytokines in the activation of the immune system. While infection is one setting in which these actions may be relevant, these data may also help to elucidate additional functional roles of interferons, such as in tumor suppression. Exploration of the transcriptional programs initiated by interferons in heterogenous and homogenous cell populations, extends our understanding of the global actions of these major mediators of inflammation, improving our comprehension of disease states, immune cell signaling cascades, the immunomodulatory mechanisms of infectious agents and potential recombinant therapies.

## Materials and Methods

### PBMC extraction and cell subset isolation

Human primary peripheral blood mononuclear cells (PBMCs) were purified from whole blood of healthy donors using Ficoll-Paque PLUS (GE Healthcare) according to manufacturer's instructions. To control for genetic variation [64], PBMCs were extracted from a single healthy donor for the cytokine comparison and dose response experiments. For the cell subset isolations where larger quantities of PBMCs were required, PBMCs were purified from buffy coat extractions from three healthy donors supplied by the Stanford University Blood Center. PBMC cell subsets (CD4^+^ and CD8^+^ T cells, B cells, NK cells and monocytes) were purified by negative selection using antibody-coated magnetic bead separation (Invitrogen) following the manufacturer's instructions. The cell subsets were isolated by negative rather than positive selection, as the consequences of cross-linking cell surface selection markers used in positive selection (CD2, CD3, CD4, CD8, CD19, CD14) on the activation state of the purified cell populations before stimulation are unknown. Cell subset purity was assessed by flow cytometry, and determined to be 67–98%.

### Cell stimulation

PBMCs and individual cell subsets were incubated at 1.5–2.0×10^6^ cells/well in RPMI 1640 medium supplemented with 10% heat-inactivated fetal calf serum and 2 mM L-glutamine (Invitrogen) at 37°C, 5% CO_2_ for 24 h before stimulation. Cells were treated with 0.6 pM recombinant IFNα2b, IFNβ1a, IFNγ, IFNω, IL12, or TNFα (R&D Systems), and sampled at time intervals from 0.5 h to 24 h after stimulation. Additionally, cells were treated with 0.1% BSA/PBS alone and used for untreated (mock) control time courses. As reference points for these time series, multiple replicates of untreated cells were sampled at time 0 (5 PBMC time zeros for the cytokine comparison, 4 PBMC time zeros for the dose response study, and 3 time zeros for each purified cell subset).

### RNA extraction and amplification

Total RNA was extracted in TRIzol LS (Invitrogen) followed by standard chloroform purification and isopropanol precipitation. RNA was re-suspended in RNase-free water, quantitated with a NanoDrop ND-1000 spectrophotometer (NanoDrop Technologies) and stored at −80°C. 500 ng total RNA was amplified using the MessageAmp modified Eberwine linear amplification procedure (Applied Biosystems). All samples to be compared were extracted and amplified together.

### Microarray analysis and data processing

4 µg amplified RNA was labeled with Cy5-dUTP (GE Healthcare) and combined with 3 µg of Cy3-labelled reference cDNA derived from a pool of RNA from a panel of 11 human cell lines (Stratagene Universal Human Reference RNA). The samples were washed and concentrated using MinElute columns (Qiagen) and competitively hybridized once to custom printed cDNA microarrays containing 37,632 elements from cDNA clones representing approximately 18,000 unique human genes (as previously described [18]). The hybridized slides were scanned using a GenePix 4000A microarray scanner (Axon Instruments). Comparative spot intensities were calculated from the images, and areas of poor quality excluded from further analysis using GenePix Pro 6.0 (Axon Instruments). The datasets discussed in this publication have been deposited in the Stanford Microarray Database [65], and NCBI's Gene Expression Omnibus [66] and are accessible through GEO Series accession number GSE17762 [67]. Analysis was restricted to cDNA elements with a regression correlation of >0.6, fluorescence intensities of >2.5 fold signal/background in Cy3 or Cy5 channels and a minimum signal intensity of >100 in both channels for at least 80% of the arrays. The expression ratios were normalized for array variation, and the data zero-transformed using a custom-designed Microsoft Excel macro (C. Liu, Stanford University) to the study-specific time zeros (average of 5 biological replicates for the cytokine comparison, 4 for the dose response study, and 3 for each purified cell subset). Time zero replicate expression profiles were highly similar with average r^2^ values >0.95. The statistical package SAM (Significance Analysis of Microarrays, version 1.15) was used to identify genes significantly differentially expressed in the normalized data sets by pair wise comparison with a minimum 2 fold cutoff at a false discovery rate of <1% of the median [68]. The transformed datasets were then hierarchically clustered using Cluster 2.11 and the results displayed using Treeview 1.60 [69]. The hypergeometric function was used to determine the significance of overlapping gene lists. Short Time-series Expression Miner (STEM) [42] was used to identify significantly represented temporal expression profiles (p<0.05 after Bonferroni multiple testing correction). The Database for Annotation, Visualization and Integrated Discovery (DAVID) [70], [71] allowed significantly enriched functional classifications of genes to be recognized. Transcription factor binding motif searching was performed using TFSearch after sequences were prepared using EZRetrieve and RepeatMasker [72]. The monocyte temporal response to IFNγ treatment was explored using Ariadne Pathway Studio 6.2 (ResNet 6 Q1 2009 Mammalian database, derived in part through text mining) mapping downstream expression and promoter binding interactions. Significantly differentially expressed genes are presented in Tables S1, S2, S3, S4, S5, S6, S7, S8.

### Quantitative RT-PCR

cDNA was synthesized from total RNA using an anchored oligo(dT)20 primer (Invitrogen) and Superscript III RT (Invitrogen), in accordance with the manufacturer's instructions. PCRs were prepared using the TaqMan Universal PCR Master Mix (Applied Biosystems), and cDNA derived from 10 ng total RNA. Relative abundance of the target transcripts was calculated by comparison to a standard curve, and normalized to the expression level of ribosomal protein L5 (RPL5). Applied Biosystems (ABI) assay IDs are as follows: CISH Hs01003603_m1; IFNGR1 Hs00988304_m1; RHOH Hs00180265_m1; RPL5 Hs00851991_u1; STAT5A Hs00559643_m1.

### Flow cytometry

The cellular composition of PBMCs and the purity of isolated PBMC cell subsets were determined by flow cytometry using a BD LSRII flow cytometer (BD Biosciences). A five color assay for CD4 (pacific blue, Molecular Probes), CD8 (FITC), CD56 (PE), CD19 (PerCP-Cy5.5), CD14 (APC-Cy7) (all BD Biosciences unless otherwise stated) was performed on cells extracted and labeled according to manufacturer's recommendations. Results were captured and analyzed using the BD FACSDiva software (BD Biosciences). The induction of apoptosis was estimated at 24 or 48 h after treatment using the Annexin V-FITC/propidium iodide detection assay (BD Biosciences).

## Supporting Information

Figure S1 The transcriptional profile of PBMCs stimulated with one of three concentrations of IFNγ. 370 genes were significantly induced by IFNγ treatment, as assessed at 0.5, 1, 2, 4, 8 and 12 h after exposure to 0.006 pM, 0.6 pM or 60 pM IFNγ (corresponding to 1, 100 or 10,000 U, respectively). The expression profiles are ordered by hierarchical clustering; the genes are displayed as rows, time points/IFNγ dose as columns. Red coloring signifies the up-regulation of expression relative to T0. The column marked S indicates (in red) which genes were significantly induced by each concentration of IFNγ.
(2.37 MB TIF)

Figure S2 Quantitative RT-PCR validation. Confirmation of the differential regulation of CISH, RHOH, IFNGR1 and STAT5A in monocytes after stimulation with IFNγ. Fold change is detailed relative to the untreated monocyte profile at 0.5, 1, 4, 8, 12 and 24 h. Relative abundance of the target transcripts was calculated by comparison to a standard curve, and normalized to the expression level of ribosomal protein L5 (RPL5). Standard deviations, calculated from triplicate samples, are marked with error bars. The corresponding transcriptional patterns of these genes derived from microarray analysis are displayed in Figure 4.
(0.55 MB TIF)

Table S1 A matrix describing the differential gene expression of PBMCs stimulated with each cytokine. The top half of the table lists the number of genes identified by SAM as significantly more highly expressed (numerator) or under-expressed (denominator) in each comparison (y axis vs. x axis) after exposure to 0.6 pM IFNα, β, ω, and γ, IL12 and TNFα. The bottom half of the matrix provides the mean correlation score (from 6 time points sampled) of the PBMC responses to each treatment compared to all other stimuli; standard deviations are marked in italics.
(0.03 MB DOC)

Table S2 PBMC cytokine activation profiles. The PBMC responses to stimulation with 0.6 pM IFNα, β, ω, and γ, IL12 and TNFα from 30 minutes to 24 h after treatment. Genes identified by SAM analysis (minimum 2 fold cutoff at a false discovery rate of <1% of the median) are identified for each cytokine compared to mock (0.1% BSA/PBS) treated PBMCs. The genes are partitioned into induced/repressed lists for each cytokine, together with mean fold expression ratios, and are ordered alphabetically using gene annotation from Source [70]. Data are summarized in Figure 1 and Table 1.
(0.18 MB XLS)

Table S3 Common response to type I interferon. 201 genes were induced after 0.6 pM treatment with IFNα, β and IFNω compared to mock (0.1% BSA/PBS) treated PBMCs. Genes identified by SAM analysis (minimum 2 fold cutoff at a false discovery rate of <1% of the median). Gene fold induction is detailed for each IFN treatment. Data are summarized in Figure 1 and Table 1.
(0.05 MB XLS)

Table S4 Genes identified to be preferentially expressed by type I or type II interferons.
(0.11 MB DOC)

Table S5 Cell-type associated genes. Genes were defined as cell type-associated if they were identified (by multiple SAM two class pairwise comparisons, FDR<1%, minimum of 2 fold change) as significantly more highly expressed in a single (unstimulated) cell type compared to all other (unstimulated) cell type gene expression profiles. Data are summarized in Figure 3a.
(0.20 MB XLS)

Table S6 The cell type-specific nature of IFNγ transcriptional programs. Purified subsets of CD4^+^ T cells, CD8^+^ T cells, B cells, NK cells and monocytes were stimulated with 0.6 pM IFNγ and sampled at 0.5, 1, 4, 8, 12 and 24 h. Genes significantly induced (determined using the SAM algorithm, minimum 2 fold cutoff at a false discovery rate of <1% of the median) by IFNγ are indicated for each purified cell subset together with mean fold expression ratios. The genes are ordered alphabetically, using gene annotation from Source [70]. Data are depicted in Figure 3b.
(0.08 MB XLS)

Table S7 The temporal transcriptional response of monocytes to 0.6 pM IFNγ. The significantly differentially expressed genes (determined using the SAM algorithm, minimum 2 fold cutoff at a false discovery rate of <1% of the median), and sampled at 0.5, 1, 4, 8, 12 and 24 h post IFNγ treatment, were separated into 7 significantly represented profiles using STEM. The table is ordered by STEM profile (as illustrated in Figure 4a), then alphabetically using gene annotation from Source [70].
(0.07 MB XLS)

Table S8 IFNγ-induced transcripts unique to mixed-cell populations. 40 genes significantly induced by IFNγ in the heterogeneous PBMC population that were not significantly induced after IFNγ treatment in any of the purified cell subsets. The genes are ordered alphabetically using gene annotation from Source [70].
(0.02 MB XLS)

## References

[1] Isaacs A, Lindenmann J (1957). Virus interference. I. The interferon.. Proc R Soc Lond B Biol Sci.

[2] Bach EA, Aguet M, Schreiber RD (1997). The IFN gamma receptor: a paradigm for cytokine receptor signaling.. Annu Rev Immunol.

[3] Kotenko SV, Gallagher G, Baurin VV, Lewis-Antes A, Shen M (2003). IFN-lambdas mediate antiviral protection through a distinct class II cytokine receptor complex.. Nat Immunol.

[4] Sheppard P, Kindsvogel W, Xu W, Henderson K, Schlutsmeyer S (2003). IL-28, IL-29 and their class II cytokine receptor IL-28R.. Nat Immunol.

[5] Pestka S, Krause CD, Walter MR (2004). Interferons, interferon-like cytokines, and their receptors.. Immunol Rev.

[6] van den Broek MF, Muller U, Huang S, Aguet M, Zinkernagel RM (1995). Antiviral defense in mice lacking both alpha/beta and gamma interferon receptors.. J Virol.

[7] Stark GR, Kerr IM, Williams BR, Silverman RH, Schreiber RD (1998). How cells respond to interferons.. Annu Rev Biochem.

[8] Sadler AJ, Williams BR (2008). Interferon-inducible antiviral effectors.. Nat Rev Immunol.

[9] Ortaldo JR, Herberman RB, Harvey C, Osheroff P, Pan YC (1984). A species of human alpha interferon that lacks the ability to boost human natural killer activity.. Proc Natl Acad Sci U S A.

[10] Hilkens CM, Schlaak JF, Kerr IM (2003). Differential responses to IFN-alpha subtypes in human T cells and dendritic cells.. J Immunol.

[11] Jaitin DA, Roisman LC, Jaks E, Gavutis M, Piehler J (2006). Inquiring into the differential action of interferons (IFNs): an IFN-alpha2 mutant with enhanced affinity to IFNAR1 is functionally similar to IFN-beta.. Mol Cell Biol.

[12] Takaoka A, Yanai H (2006). Interferon signalling network in innate defence.. Cell Microbiol.

[13] Baig E, Fish EN (2008). Distinct signature type I interferon responses are determined by the infecting virus and the target cell.. Antivir Ther.

[14] Boehm U, Klamp T, Groot M, Howard JC (1997). Cellular responses to interferon-gamma.. Annu Rev Immunol.

[15] Newport MJ, Huxley CM, Huston S, Hawrylowicz CM, Oostra BA (1996). A mutation in the interferon-gamma-receptor gene and susceptibility to mycobacterial infection.. N Engl J Med.

[16] Dunn GP, Koebel CM, Schreiber RD (2006). Interferons, immunity and cancer immunoediting.. Nat Rev Immunol.

[17] Schiepers OJ, Wichers MC, Maes M (2005). Cytokines and major depression.. Prog Neuropsychopharmacol Biol Psychiatry.

[18] Alizadeh AA, Eisen MB, Davis RE, Ma C, Lossos IS (2000). Distinct types of diffuse large B-cell lymphoma identified by gene expression profiling [see comments].. Nature.

[19] Lock C, Hermans G, Pedotti R, Brendolan A, Schadt E (2002). Gene-microarray analysis of multiple sclerosis lesions yields new targets validated in autoimmune encephalomyelitis.. Nat Med.

[20] Griffiths MJ, Shafi MJ, Popper SJ, Hemingway CA, Kortok MM (2005). Genomewide analysis of the host response to malaria in Kenyan children.. J Infect Dis.

[21] Popper SJ, Shimizu C, Shike H, Kanegaye JT, Newburger JW (2007). Gene-expression patterns reveal underlying biological processes in Kawasaki disease.. Genome Biol.

[22] Ramilo O, Allman W, Chung W, Mejias A, Ardura M (2007). Gene expression patterns in blood leukocytes discriminate patients with acute infections.. Blood.

[23] Jacobsen M, Repsilber D, Gutschmidt A, Neher A, Feldmann K (2007). Candidate biomarkers for discrimination between infection and disease caused by Mycobacterium tuberculosis.. J Mol Med.

[24] Watson MA, Ylagan LR, Trinkaus KM, Gillanders WE, Naughton MJ (2007). Isolation and molecular profiling of bone marrow micrometastases identifies TWIST1 as a marker of early tumor relapse in breast cancer patients.. Clin Cancer Res.

[25] Rubins KH, Hensley LE, Jahrling PB, Whitney AR, Geisbert TW (2004). The host response to smallpox: analysis of the gene expression program in peripheral blood cells in a nonhuman primate model.. Proc Natl Acad Sci U S A.

[26] Simmons CP, Popper S, Dolocek C, Chau TN, Griffiths M (2007). Patterns of host genome-wide gene transcript abundance in the peripheral blood of patients with acute dengue hemorrhagic fever.. J Infect Dis.

[27] Zimmerer JM, Lesinski GB, Ruppert AS, Radmacher MD, Noble C (2008). Gene expression profiling reveals similarities between the in vitro and in vivo responses of immune effector cells to IFN-alpha.. Clin Cancer Res.

[28] Jenner RG, Young RA (2005). Insights into host responses against pathogens from transcriptional profiling.. Nat Rev Microbiol.

[29] Der SD, Zhou A, Williams BR, Silverman RH (1998). Identification of genes differentially regulated by interferon alpha, beta, or gamma using oligonucleotide arrays.. Proc Natl Acad Sci U S A.

[30] Dolken L, Ruzsics Z, Radle B, Friedel CC, Zimmer R (2008). High-resolution gene expression profiling for simultaneous kinetic parameter analysis of RNA synthesis and decay.. RNA.

[31] Sana TR, Janatpour MJ, Sathe M, McEvoy LM, McClanahan TK (2005). Microarray analysis of primary endothelial cells challenged with different inflammatory and immune cytokines.. Cytokine.

[32] Hartman SE, Bertone P, Nath AK, Royce TE, Gerstein M (2005). Global changes in STAT target selection and transcription regulation upon interferon treatments.. Genes Dev.

[33] Darnell JE, Kerr IM, Stark GR (1994). Jak-STAT pathways and transcriptional activation in response to IFNs and other extracellular signaling proteins.. Science.

[34] Platanias LC (2005). Mechanisms of type-I- and type-II-interferon-mediated signalling.. Nat Rev Immunol.

[35] Brinkmann V, Geiger T, Alkan S, Heusser CH (1993). Interferon alpha increases the frequency of interferon gamma-producing human CD4+ T cells.. J Exp Med.

[36] de Jong R, Altare F, Haagen IA, Elferink DG, Boer T (1998). Severe mycobacterial and Salmonella infections in interleukin-12 receptor-deficient patients.. Science.

[37] Wajant H, Pfizenmaier K, Scheurich P (2003). Tumor necrosis factor signaling.. Cell Death Differ.

[38] Kempe S, Kestler H, Lasar A, Wirth T (2005). NF-kappaB controls the global pro-inflammatory response in endothelial cells: evidence for the regulation of a pro-atherogenic program.. Nucleic Acids Res.

[39] Palmer C, Diehn M, Alizadeh AA, Brown PO (2006). Cell-type specific gene expression profiles of leukocytes in human peripheral blood.. BMC Genomics.

[40] Holling TM, Schooten E, van den Elsen PJ (2004). Function and regulation of MHC class II molecules in T-lymphocytes: of mice and men.. Hum Immunol.

[41] Ramsey SA, Klemm SL, Zak DE, Kennedy KA, Thorsson V (2008). Uncovering a macrophage transcriptional program by integrating evidence from motif scanning and expression dynamics.. PLoS Comput Biol.

[42] Ernst J, Bar-Joseph Z (2006). STEM: a tool for the analysis of short time series gene expression data.. BMC Bioinformatics.

[43] Vasanwala FH, Kusam S, Toney LM, Dent AL (2002). Repression of AP-1 function: a mechanism for the regulation of Blimp-1 expression and B lymphocyte differentiation by the B cell lymphoma-6 protooncogene.. J Immunol.

[44] Dimitriou ID, Clemenza L, Scotter AJ, Chen G, Guerra FM (2008). Putting out the fire: coordinated suppression of the innate and adaptive immune systems by SOCS1 and SOCS3 proteins.. Immunol Rev.

[45] Ram PA, Waxman DJ (2000). Role of the cytokine-inducible SH2 protein CIS in desensitization of STAT5b signaling by continuous growth hormone.. J Biol Chem.

[46] Chae HD, Lee KE, Williams DA, Gu Y (2008). Cross-talk between RhoH and Rac1 in regulation of actin cytoskeleton and chemotaxis of hematopoietic progenitor cells.. Blood.

[47] Daryadel A, Yousefi S, Troi D, Schmid I, Schmidt-Mende J (2009). RhoH/TTF negatively regulates leukotriene production in neutrophils.. J Immunol.

[48] Ogilvie RL, Sternjohn JR, Rattenbacher B, Vlasova IA, Williams DA (2009). Tristetraprolin Mediates Interferon-{gamma} mRNA Decay.. J Biol Chem.

[49] Kovarik P, Sauer I, Schaljo B (2007). Molecular mechanisms of the anti-inflammatory functions of interferons.. Immunobiology.

[50] LeibundGut-Landmann S, Waldburger JM, Krawczyk M, Otten LA, Suter T (2004). Mini-review: Specificity and expression of CIITA, the master regulator of MHC class II genes.. Eur J Immunol.

[51] Rubins KH, Hensley LE, Wahl-Jensen V, Daddario DiCaprio KM, Young HA (2007). The temporal program of peripheral blood gene expression in the response of nonhuman primates to Ebola hemorrhagic fever.. Genome Biol.

[52] Pathan N, Hemingway CA, Alizadeh AA, Stephens AC, Boldrick JC (2004). Role of interleukin 6 in myocardial dysfunction of meningococcal septic shock.. Lancet.

[53] Popper SJ, Watson VE, Shimizu C, Kanegaye JT, Burns JC (2009). Gene transcript abundance profiles distinguish Kawasaki disease from adenovirus infection.. J Infect Dis.

[54] Schlaak JF, Hilkens CM, Costa-Pereira AP, Strobl B, Aberger F (2002). Cell-type and donor-specific transcriptional responses to interferon-alpha. Use of customized gene arrays.. J Biol Chem.

[55] Whitney AR, Diehn M, Popper SJ, Alizadeh AA, Boldrick JC (2003). Individuality and variation in gene expression patterns in human blood.. Proc Natl Acad Sci U S A.

[56] Boldrick JC, Alizadeh AA, Diehn M, Dudoit S, Liu CL (2002). Stereotyped and specific gene expression programs in human innate immune responses to bacteria.. Proc Natl Acad Sci U S A.

[57] Thompson LJ, Dunstan SJ, Dolecek C, Perkins T, House D (2009). Transcriptional response in the peripheral blood of patients infected with Salmonella enterica serovar Typhi.. Proc Natl Acad Sci U S A.

[58] Aberger F, Costa-Pereira AP, Schlaak JF, Williams TM, O'Shaughnessy RF (2001). Analysis of gene expression using high-density and IFN-gamma-specific low-density cDNA arrays.. Genomics.

[59] Indraccolo S, Pfeffer U, Minuzzo S, Esposito G, Roni V (2007). Identification of genes selectively regulated by IFNs in endothelial cells.. J Immunol.

[60] Kalveram B, Schmidtke G, Groettrup M (2008). The ubiquitin-like modifier FAT10 interacts with HDAC6 and localizes to aggresomes under proteasome inhibition.. J Cell Sci.

[61] Lukasiak S, Schiller C, Oehlschlaeger P, Schmidtke G, Krause P (2008). Proinflammatory cytokines cause FAT10 upregulation in cancers of liver and colon.. Oncogene.

[62] Radomska HS, Huettner CS, Zhang P, Cheng T, Scadden DT (1998). CCAAT/enhancer binding protein alpha is a regulatory switch sufficient for induction of granulocytic development from bipotential myeloid progenitors.. Mol Cell Biol.

[63] Nerlov C (2004). C/EBPalpha mutations in acute myeloid leukaemias.. Nat Rev Cancer.

[64] Dimas AS, Deutsch S, Stranger BE, Montgomery SB, Borel C (2009). Common regulatory variation impacts gene expression in a cell type-dependent manner.. Science.

[65] Stanford Microarray Database.. http://genome-www5.stanford.edu.

[66] Edgar R, Domrachev M, Lash AE (2002). Gene Expression Omnibus: NCBI gene expression and hybridization array data repository.. Nucleic Acids Res.

[67] Gene Expression Omnibus Accession Number GSE17762. Available: http://www.ncbi.nlm.nih.gov/geo/query/acc.cgi?acc=GSE17762

[68] Tusher VG, Tibshirani R, Chu G (2001). Significance analysis of microarrays applied to the ionizing radiation response.. Proc Natl Acad Sci U S A.

[69] Eisen MB, Spellman PT, Brown PO, Botstein D (1998). Cluster analysis and display of genome-wide expression patterns.. Proc Natl Acad Sci U S A.

[70] Dennis G, Sherman BT, Hosack DA, Yang J, Gao W (2003). DAVID: Database for Annotation, Visualization, and Integrated Discovery.. Genome Biol.

[71] Huang dW, Sherman BT, Lempicki RA (2009). Systematic and integrative analysis of large gene lists using DAVID bioinformatics resources.. Nat Protoc.

[72] Heinemeyer T, Wingender E, Reuter I, Hermjakob H, Kel AE (1998). Databases on transcriptional regulation: TRANSFAC, TRRD and COMPEL.. Nucleic Acids Res.

[73] Diehn M, Sherlock G, Binkley G, Jin H, Matese JC (2003). SOURCE: a unified genomic resource of functional annotations, ontologies, and gene expression data.. Nucleic Acids Res.

