# Supplementary figures and images for: Dissecting Interferon-Induced Transcriptional Programs in Human Peripheral Blood Cells
Source: PLoS One. 2010 Mar 22;5(3):e9753. doi: 10.1371/journal.pone.0009753 (PMC2842296; doi:10.1371/journal.pone.0009753)

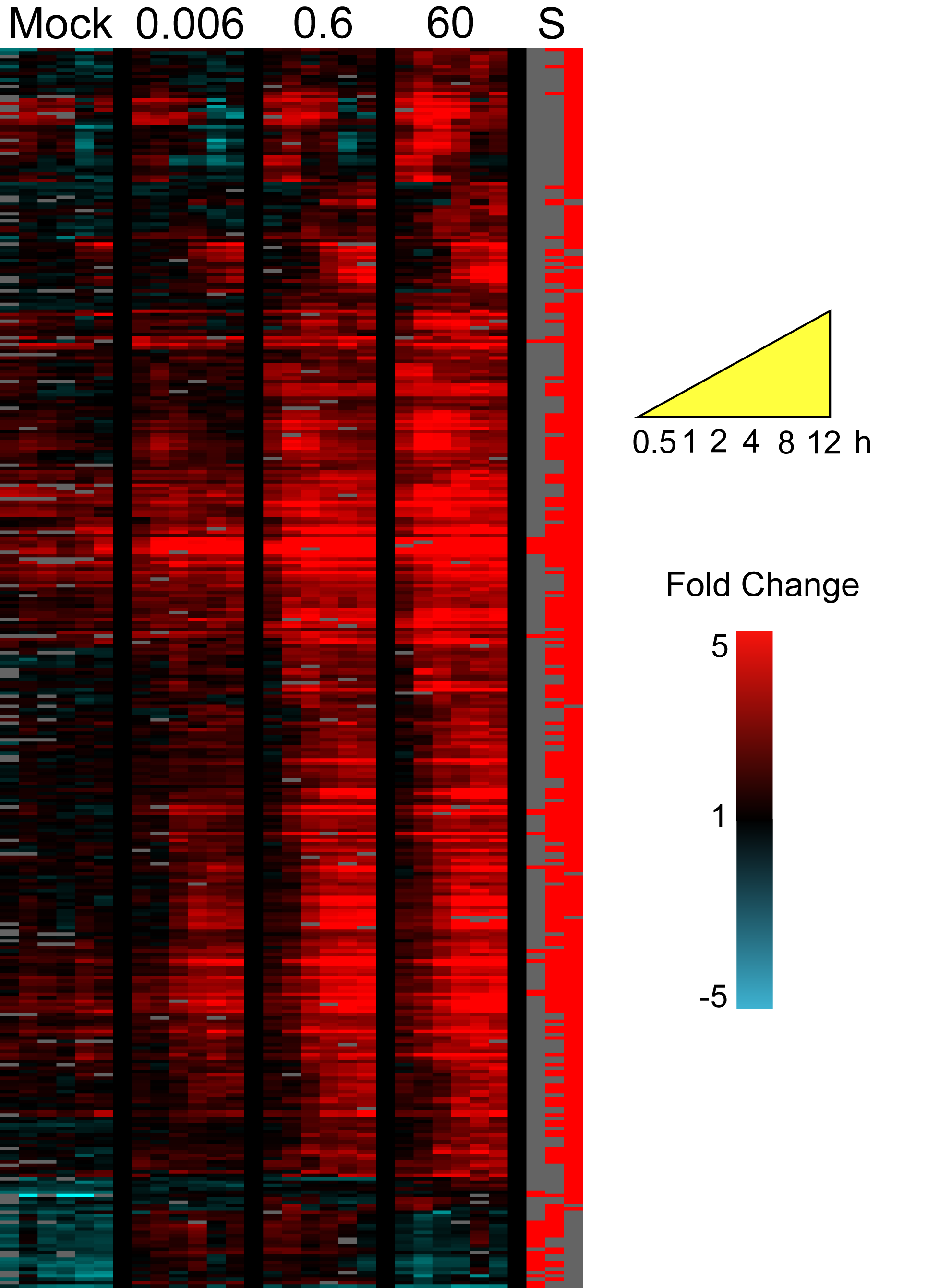

Supplement: Figure S1 — The transcriptional profile of PBMCs stimulated with one of three concentrations of IFNγ. 370 genes were significantly induced by IFNγ treatment, as assessed at 0.5, 1, 2, 4, 8 and 12 h after exposure to 0.006 pM, 0.6 pM or 60 pM IFNγ (corresponding to 1, 100 or 10,000 U, respectively). The expression profiles are ordered by hierarchical clustering; the genes are displayed as rows, time points/IFNγ dose as columns. Red coloring signifies the up-regulation of expression relative to T0. The column marked S indicates (in red) which genes were significantly induced by each concentration of IFNγ. (2.37 MB TIF) [file pone.0009753.s001.tif]

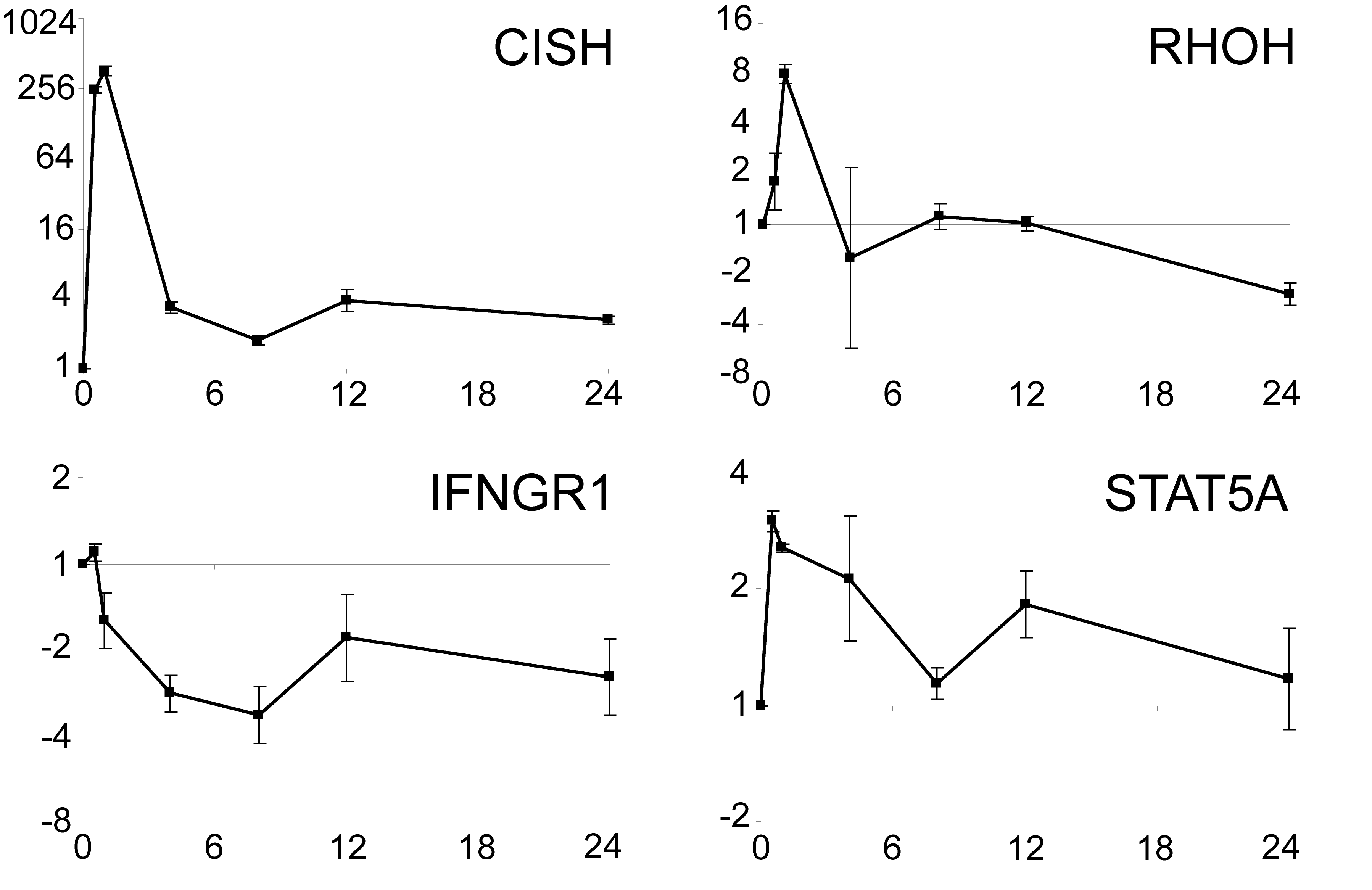

Supplement: Figure S2 — Quantitative RT-PCR validation. Confirmation of the differential regulation of CISH, RHOH, IFNGR1 and STAT5A in monocytes after stimulation with IFNγ. Fold change is detailed relative to the untreated monocyte profile at 0.5, 1, 4, 8, 12 and 24 h. Relative abundance of the target transcripts was calculated by comparison to a standard curve, and normalized to the expression level of ribosomal protein L5 (RPL5). Standard deviations, calculated from triplicate samples, are marked with error bars. The corresponding transcriptional patterns of these genes derived from microarray analysis are displayed in Figure 4. (0.55 MB TIF) [file pone.0009753.s002.tif]
