# Supplementary material for: Dissecting Interferon-Induced Transcriptional Programs in Human Peripheral Blood Cells
Source: PLoS One. 2010 Mar 22;5(3):e9753. doi: 10.1371/journal.pone.0009753 (PMC2842296; doi:10.1371/journal.pone.0009753)
Supplement: Table S1 — A matrix describing the differential gene expression of PBMCs stimulated with each cytokine. The top half of the table lists the number of genes identified by SAM as significantly more highly expressed (numerator) or under-expressed (denominator) in each comparison (y axis vs. x axis) after exposure to 0.6 pM IFNα, β, ω, and γ, IL12 and TNFα. The bottom half of the matrix provides the mean correlation score (from 6 time points sampled) of the PBMC responses to each treatment compared to all other stimuli; standard deviations are marked in italics. (0.03 MB DOC) [file pone.0009753.s003.doc]

### Table S1 (Waddell *et al*.)

|  | **Mock** | **IFNα** | **IFNβ** | **IFNω** | **IFNγ** | **IL12** | **TNFα** |
| --- | --- | --- | --- | --- | --- | --- | --- |
| **Mock** | **X** | **226**  **31** | **370**  **114** | **288**  **89** | **111**  **77** | **57**  **53** | **130**  **125** |
| **IFNα** | **0.28**  ***(0.16)*** | **X** | **28**  **82** | **5**  **22** | **44**  **177** | **55**  **220** | **126**  **259** |
| **IFNβ** | **0.19**  ***(0.07)*** | **0.69**  ***(0.15)*** | **X** | **2**  **3** | **28**  **210** | **17**  **249** | **162**  **338** |
| **IFNω** | **0.17**  ***(0.10)*** | **0.68**  ***(0.24)*** | **0.77**  ***(0.24)*** | **X** | **18**  **151** | **32**  **192** | **88**  **257** |
| **IFNγ** | **0.21**  ***(0.22)*** | **0.42**  ***(0.17)*** | **0.50**  ***(0.14)*** | **0.54**  ***(0.11)*** | **X** | **3**  **2** | **58**  **61** |
| **IL12** | **0.28**  ***(0.16)*** | **0.28**  ***(0.17)*** | **0.43**  ***(0.11)*** | **0.33**  ***(0.18)*** | **0.56**  ***(0.19)*** | **X** | **63**  **50** |
| **TNFα** | **0.03**  ***(0.16)*** | **0.16**  ***(0.11)*** | **0.22**  ***(0.13)*** | **0.27**  ***(0.09)*** | **0.35**  ***(0.08)*** | **0.35**  **(*0.10)*** | **X** |
