# Supplementary material for: Dissecting Interferon-Induced Transcriptional Programs in Human Peripheral Blood Cells
Source: PLoS One. 2010 Mar 22;5(3):e9753. doi: 10.1371/journal.pone.0009753 (PMC2842296; doi:10.1371/journal.pone.0009753)
Supplement: Table S4 — Genes identified to be preferentially expressed by type I or type II interferons. (0.11 MB DOC) [file pone.0009753.s006.doc]

Table S4. Genes identified to be preferentially expressed by type I or type II interferons.

| Genes preferentially induced by type I IFNs | | **Mean Fold Change** | | |
| --- | --- | --- | --- | --- |
| **Gene ID** | Putative Gene Function | **IFNα** | **IFNβ** | **IFNω** |
| ADM | Adrenomedullin | 3.2 | 2.8 | 3.4 |
| AGT | Serine (or cysteine) proteinase inhibitor | 25.2 | 31.7 | 22.1 |
| AKAP2 | A kinase (PRKA) anchor protein 2 | 2.9 | 3.3 | 2.9 |
| ATF3 | Activating transcription factor 3 | 2.4 | 2.8 | 2.4 |
| BAG1 | BCL2-associated athanogene | 2.1 | 2.9 | 2.3 |
| BF | B-factor, properdin | 3.3 | 3.4 | 2.5 |
| BLZF1 | Basic leucine zipper nuclear factor 1 | 2.2 | 3.0 | 2.3 |
| BRCA1 | Breast cancer 1, early onset | 3.4 | 3.7 | 3.4 |
| BRDG1 | BCR downstream signalling 1 | 2.8 | 2.9 | 2.1 |
| BST2 | Bone marrow stromal cell antigen 2 | 2.4 | 2.9 | 2.9 |
| C1QA | Complement component 1, alpha polypeptide | 3.6 | 4.0 | 3.3 |
| CAMK2A | Calcium/calmodulin-dependent protein kinase | 2.2 | 2.3 | 2.1 |
| CBR1 | Carbonyl reductase 1 | 2.4 | 2.8 | 2.1 |
| CCL4 | Small inducible cytokine A4 | 2.0 | 2.4 | 2.1 |
| CCL7 | Small inducible cytokine A7 | 2.8 | 2.6 | 2.6 |
| CCNA1 | Cyclin A1 | 2.9 | 2.7 | 2.8 |
| CD38 | CD38 antigen (p45) | 2.5 | 3.1 | 2.3 |
| CD69 | CD69 antigen (early T-cell activation antigen) | 3.4 | 3.9 | 3.3 |
| COL3A1 | Collagen, type III, alpha 1 | 3.3 | 3.8 | 3.4 |
| CXCL11 | Small inducible cytokine subfamily B | 4.8 | 6.7 | 5.0 |
| CYP2J2 | Cytochrome P450, subfamily IIJ polypeptide 2 | 3.4 | 2.8 | 2.6 |
| DEFB1 | Defensin, beta 1 | 7.9 | 8.4 | 5.6 |
| DJ742C19.2 | Phorbolin (similar to apolipoprotein B) | 3.1 | 3.3 | 3.7 |
| DSC2 | Desmocollin 2 | 2.5 | 2.7 | 2.2 |
| DUSP7 | Dual specificity phosphatase 7 | 2.5 | 2.9 | 2.7 |
| E2-EPF | Ubiquitin carrier protein | 2.1 | 2.5 | 2.2 |
| ECE1 | Endothelin converting enzyme 1 | 2.5 | 3.0 | 2.7 |
| EIF2AK2 | Protein kinase, interferon-inducible | 4.6 | 5.0 | 5.5 |
| EIF4ENIF1 | EIF4E-transporter | 2.2 | 3.2 | 2.9 |
| ELF1 | E74-like factor 1 (ets transcription factor) | 2.4 | 2.3 | 2.2 |
| FANCA | Fanconi anemia, complementation group A | 2.3 | 2.4 | 2.2 |
| FASLG | Tumor necrosis factor superfamily, member 6 | 2.1 | 3.0 | 2.3 |
| FGF1 | Fibroblast growth factor 1 (acidic) | 2.3 | 3.0 | 2.2 |
| FLJ10217 | Oxysterol-binding protein-related protein 1 | 2.4 | 3.2 | 3.0 |
| FLNA | Filamin A, alpha (actin-binding protein-280) | 2.7 | 2.4 | 2.5 |
| FUT4 | Fucosyltransferase 4 | 2.5 | 3.3 | 2.6 |
| G1P3 | Interferon, alpha-inducible protein | 2.8 | 2.4 | 2.1 |
| GMPR | Guanosine monophosphate reductase | 9.9 | 11.8 | 20.9 |
| GNA14 | Guanine nucleotide binding protein, alpha 14 | 2.2 | 2.1 | 2.2 |
| GZMB | Granzyme B | 2.7 | 2.5 | 2.5 |
| HIST2H2AA | H2A histone family, member O | 2.1 | 2.1 | 2.1 |
| HOMER2 | Homer, neuronal immediate early gene, 2 | 2.5 | 3.0 | 2.4 |
| HS6ST | Heparan sulfate 6-O-sulfotransferase | 3.2 | 3.2 | 3.3 |
| HSPA1A | Heat shock 70kD protein 1A | 2.1 | 3.1 | 2.4 |
| HSXIAPAF1 | XIAP associated factor-1 | 2.7 | 3.0 | 2.5 |
| IFI16 | Interferon, gamma-inducible protein 16 | 2.2 | 2.6 | 2.3 |
| IFI27 | Interferon, alpha-inducible protein 27 | 8.7 | 7.9 | 6.3 |
| IFI35 | Interferon-induced protein 35 | 3.0 | 3.3 | 2.7 |
| IFI75 | Interferon-induced protein 75, 52kD | 2.0 | 2.4 | 2.1 |
| IFIT1 | Interferon-induced protein | 27.3 | 13.6 | 30.7 |
| IFITM1 | Interferon induced transmembrane protein 1 | 3.9 | 4.3 | 3.9 |
| IFITM2 | Interferon induced transmembrane protein 2 | 4.5 | 5.2 | 4.9 |
| IFITM3 | Interferon induced transmembrane protein 3 | 3.9 | 3.5 | 4.1 |
| IFRG28 | 28kD interferon responsive protein | 5.3 | 8.5 | 5.6 |
| IL1R2 | Interleukin 1 receptor, type II | 2.4 | 2.3 | 2.0 |
| IL1RN | Interleukin 1 receptor antagonist | 9.5 | 10.2 | 10.0 |
| IL6 | Interleukin 6 (interferon, beta 2) | 2.4 | 2.2 | 2.3 |
| IL8RB | Interleukin 8 receptor, beta | 2.1 | 2.7 | 2.3 |
| IRF2 | Interferon regulatory factor 2 | 12.7 | 12.2 | 13.2 |
| IRF7 | Interferon regulatory factor 7 | 2.5 | 2.5 | 3.0 |
| ISG15 | Interferon-stimulated protein, 15 kDa | 11.9 | 13.7 | 12.1 |
| ISG20 | Interferon stimulated gene (20kD) | 4.4 | 5.0 | 4.4 |
| JUP | Junction plakoglobin | 2.5 | 3.0 | 2.9 |
| KDELR2 | Endoplasmic reticulum receptor 2 | 2.5 | 3.0 | 2.7 |
| KLF6 | Core promoter element binding protein | 2.1 | 2.5 | 2.2 |
| LAG3 | Lymphocyte-activation gene 3 | 2.9 | 3.4 | 2.8 |
| LGALS3BP | Lectin, galactoside-binding, 3 binding protein | 2.5 | 2.9 | 2.5 |
| LGALS9 | Lectin, galactoside-binding, soluble, 9 | 2.1 | 2.4 | 2.0 |
| LGMN | Protease, cysteine, 1 (legumain) | 2.2 | 2.2 | 3.4 |
| LMO2 | LIM domain only 2 (rhombotin-like 1) | 2.3 | 3.5 | 2.6 |
| LOC54453 | Ras association domain containing protein | 2.3 | 2.4 | 2.5 |
| LY6E | Lymphocyte antigen 6 complex, locus E | 5.0 | 4.7 | 4.2 |
| MAP2K5 | Mitogen-activated protein kinase kinase 5 | 2.7 | 2.8 | 2.5 |
| MNDA | Myeloid cell nuclear differentiation antigen | 2.9 | 3.8 | 3.0 |
| MPHOSPH1 | M-phase phosphoprotein 1 | 2.3 | 3.4 | 2.7 |
| MS4A7 | Membrane-spanning 4-domains, member 7 | 6.1 | 2.9 | 2.4 |
| MTAP44 | Interferon-induced microtubular protein | 2.1 | 3.6 | 4.2 |
| MX1 | Myxovirus (influenza) resistance 1 | 5.3 | 9.1 | 7.6 |
| MX2 | Myxovirus (influenza) resistance 2 | 7.3 | 9.1 | 8.7 |
| NR3C1 | Nuclear receptor subfamily 3, group C | 3.3 | 4.3 | 3.5 |
| OAS1 | 2'-5'-oligoadenylate synthetase 1 | 3.1 | 3.3 | 3.1 |
| OAS2 | 2'-5'-oligoadenylate synthetase 2 | 3.2 | 4.7 | 3.8 |
| OAS3 | 2'-5'-oligoadenylate synthetase 3 | 6.7 | 8.5 | 8.7 |
| OLIG2 | Protein kinase C binding protein 2 | 2.7 | 2.4 | 2.1 |
| P8 | P8 protein (candidate of metastasis 1) | 11.2 | 19.9 | 13.8 |
| PDGFB | Platelet-derived growth factor | 2.2 | 2.7 | 2.5 |
| PDGFRL | Platelet-derived growth factor receptor-like | 12.6 | 7.9 | 3.9 |
| PKD2 | Protein kinase D2 | 2.1 | 2.4 | 2.3 |
| PLSCR1 | Phospholipid scramblase 1 | 3.0 | 3.5 | 4.0 |
| PMAIP1 | Phorbol-induced protein 1 | 2.1 | 2.1 | 2.1 |
| PML | Promyelocytic leukemia | 2.6 | 3.1 | 2.5 |
| PPARBP | PPAR binding protein | 2.1 | 2.7 | 2.4 |
| RGS1 | Regulator of G-protein signaling 1 | 7.5 | 4.3 | 2.8 |
| RGS6 | Regulator of G-protein signaling 6 | 2.4 | 2.5 | 2.3 |
| RI58 | Retinoic acid- and interferon-inducible protein | 3.2 | 4.4 | 3.4 |
| RNF21 | Ring finger protein 21, interferon-responsive | 2.1 | 2.4 | 2.1 |
| SFRS10 | Splicing factor, arginine/serine-rich 10 | 2.7 | 3.2 | 2.9 |
| SP100 | Nuclear antigen | 2.4 | 2.9 | 2.6 |
| SP110 | Interferon-induced protein 41 | 2.2 | 2.8 | 2.4 |
| SP140 | Nuclear body protein | 3.3 | 2.6 | 2.6 |
| SPIB | Spi-B transcription factor (Spi-1/PU.1 related) | 4.6 | 6.4 | 6.0 |
| SUPT3H | Suppressor of Ty (S.cerevisiae) 3 homolog | 2.5 | 2.8 | 2.4 |
| TARBP1 | TAR (HIV) RNA-binding protein 1 | 3.0 | 4.0 | 3.5 |
| TCN2 | Transcobalamin II; macrocytic anemia | 2.4 | 2.6 | 2.3 |
| TCTEL1 | T-complex-associated-testis-expressed | 2.5 | 3.1 | 2.4 |
| TGM1 | Transglutaminase 1 | 7.7 | 5.9 | 5.1 |
| TLR3 | Toll-like receptor 3 | 3.2 | 4.4 | 2.7 |
| TNFSF10 | Tumor necrosis factor superfamily, 10 | 2.5 | 3.4 | 3.3 |
| TOR1B | Torsin family 1, member B (torsin B) | 2.7 | 3.4 | 3.0 |
| TRIM22 | Stimulated trans-acting factor | 2.1 | 2.9 | 2.2 |
| UNC93B1 | unc93 (C.elegans) homolog B | 2.2 | 2.7 | 2.8 |
| USP18 | Ubiquitin specific protease 18 | 12.4 | 13.8 | 12.8 |
| WASPIP | Wiskott-Aldrich syndrome interacting protein | 2.5 | 2.0 | 2.3 |
| XAP4 | HBV associated factor | 2.2 | 2.4 | 2.1 |
|  | | | | |
| **Genes preferentially induced by IFNγ** | | Mean Fold Change | | |
| **Gene ID** | Putative Gene Function | **IFNα** | **IFNβ** | **IFNω** |
| ASPH | Aspartate beta-hydroxylase | 2.1 | 2.0 | 2.3 |
| C/EBPα | CCAAT/enhancer binding protein (C/EBP) | 2.1 | 2.2 | 2.3 |
| CLECSF14 | Macrophage lectin 2 (calcium dependent) | 2.6 | 3.0 | 4.1 |
| CXCL9 | Monokine induced by gamma interferon | 5.5 | 4.0 | 4.5 |
| FCGR1A | Fc fragment of IgG | 3.6 | 2.5 | 3.8 |
| IL6R | Interleukin 6 receptor | 2.4 | 2.4 | 2.7 |
| LIMK2 | LIM domain kinase 2 | 2.4 | 2.3 | 2.1 |
| MHC2TA | MHC class II transactivator | 2.2 | 2.1 | 2.3 |
| SLC1A5 | Solute carrier family 1, member 5 | 3.2 | 2.2 | 2.4 |
| UBD | Diubiquitin | 2.8 | 3.5 | 3.8 |

PBMCs were treated with 0.6 pM IFNα, β, ω, and γ and sampled at time intervals of 0.5, 1, 4, 8, 12 and 24 h. A comparison of IFNα, β, and ω and IFNγ PBMC activation profiles identified 114 genes to be significantly more highly expressed by type I IFNs, and 10 genes to be more highly expressed by IFNγ treatment. The mean fold differences between IFNα, β, and ω and IFNγ PBMC responses are marked. The genes are ordered alphabetically, using annotation from Source [70].
